# How experts and novices judge other people’s knowledgeability from language use

**DOI:** 10.3758/s13423-023-02433-9

**Published:** 2024-01-04

**Authors:** Alexander H. Bower, Nicole Han, Ansh Soni, Miguel P. Eckstein, Mark Steyvers

**Affiliations:** 1 Department of Cognitive Sciences, University of California, Irvine, CA USA; 2 Department of Psychological and Brain Sciences, University of California, Santa Barbara, CA USA; 3 Department of Electrical and Computer Engineering, University of California, Santa Barbara, CA USA; 4 Department of Computer Science, University of California, Santa Barbara, CA USA; 5 Institute for Collaborative Biotechnologies, University of California, Santa Barbara, CA USA

**Keywords:** Theory of mind, Language, Decision-making, Mindreading, Knowledge

## Abstract

How accurate are people in judging someone else’s knowledge based on their language use, and do more knowledgeable people use different cues to make these judgments? We address this by recruiting a group of participants (“informants”) to answer general knowledge questions and describe various images belonging to different categories (e.g., cartoons, basketball). A second group of participants (“evaluators”) also answer general knowledge questions and decide who is more knowledgeable within pairs of informants, based on these descriptions. Evaluators perform above chance at identifying the most knowledgeable informants (65% with only one description available). The less knowledgeable evaluators base their decisions on the number of specific statements, regardless of whether the statements are true or false. The more knowledgeable evaluators treat true and false statements differently and penalize the knowledge they attribute to informants who produce specific yet false statements. Our findings demonstrate the power of a few words when assessing others’ knowledge and have implications for how misinformation is processed differently between experts and novices.

**Supplementary Information:**

The online version contains supplementary material available at 10.3758/s13423-023-02433-9.

## Introduction

Can just a few words reveal how much someone knows about a subject? Many situations in daily life require us to make quick assessments of others’ knowledge. Perhaps we ask a friend for a wine recommendation at a new restaurant or a stranger for directions in an unfamiliar town. How well can we infer the extent and validity of their knowledge based solely on the language that they use, especially if we lack their lexicon? Using our wine example: say your friend recommends an “attractive Zinfandel with a beefy character and a complex chewiness, complemented by its dusty finish”? The specificity of these terms may bolster their authority, expose their ignorance, or simply baffle you.

**Fig. 1:**
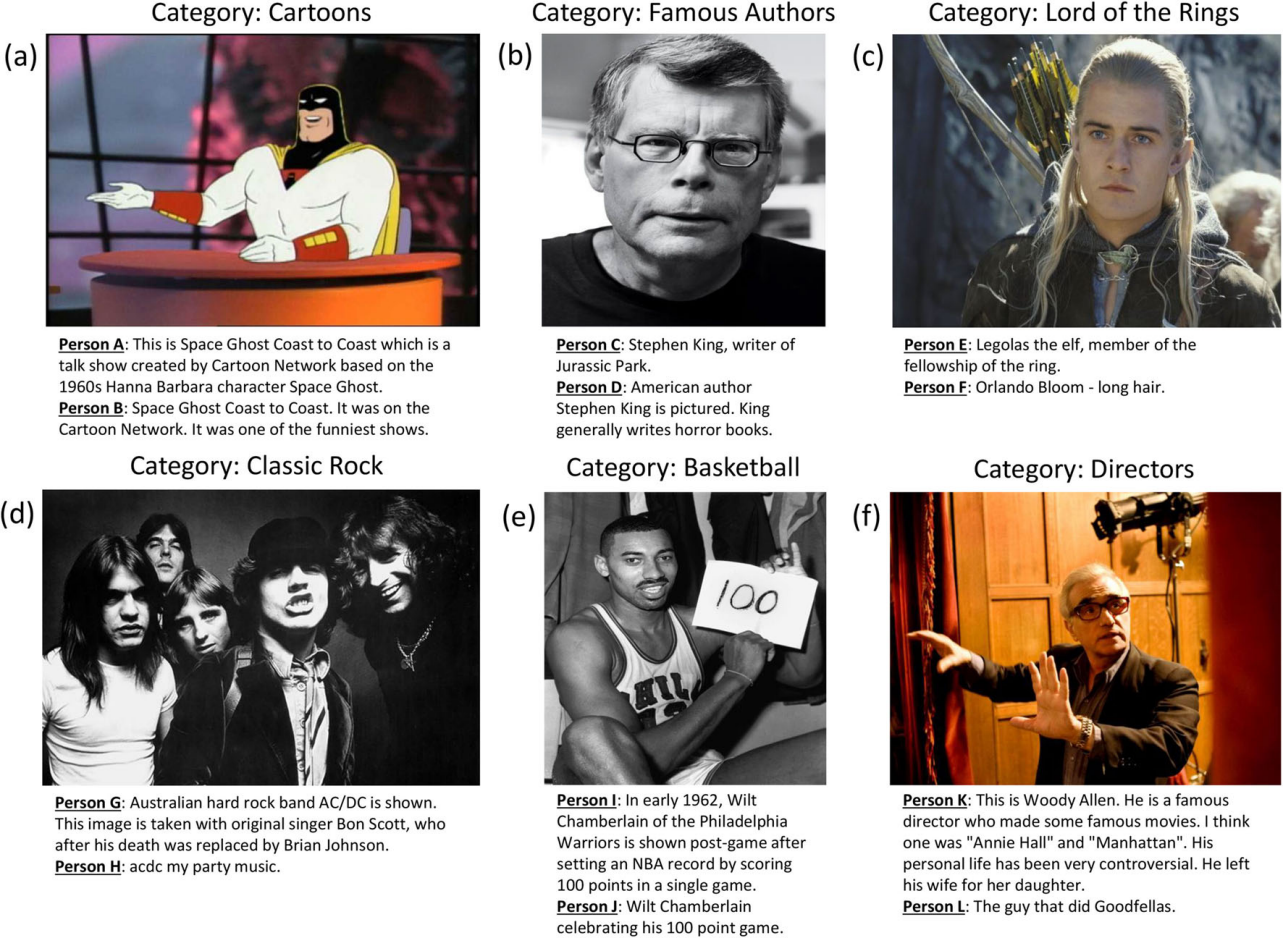
Illustration of the informant discrimination task in which the goal is to identify the most knowledgeable of two informants based on their image descriptions. Panels (**a**)–(**f**) show different example image stimuli and pairs of informant descriptions. The most knowledgeable informants of each pair, as assessed by the general knowledge questions, are A, D, E, G, I, and L

Not all situations are so innocuous, however. Being able to quickly and accurately discern truth from falsehood in brief exchanges has become increasingly vital as misinformation (and disinformation) pollutes social media (Suarez-Lledo & Alvarez-Galvez, 2021; Di Domenico, Sit, Ishizaka, & Nunan, 2021; Kouzy et al., 2020). While there are proposed individual-level explanations for why people fall victim to such “fake news” (e.g., Pennycook & Rand, 2021), less is known about how the linguistic content of misinformation *itself* deceives naive users. This challenge is further exacerbated by the fact that social media posts are rarely consumed in isolation. Rather, users are routinely bombarded by hundreds of competing posts from varying sources. How do users determine which are reputable in these complex, information-dense environments? Exploring how surface-level features of text influence people’s perceptions of credibility can help us better understand how misinformation is propagated and can inform efforts to improve media literacy (e.g., Vraga, Bode, & Tully, 2022).

To demonstrate this inferential challenge, consider Fig. 1, which features examples of stimuli and responses from our current study. In it are images depicting various domains: cartoons, famous authors, *The Lord of the Rings*, classic rock, basketball, and directors, respectively. Below each image are two people’s descriptions of the content depicted. Can you tell, based on those descriptions, who the more knowledgeable person is? Perhaps you know a lot about one of these domains, so this distinction is easy. But what if you lack this knowledge? How can you tell who knows more? Do you find yourself relying on a particular heuristic, such as the number of details mentioned and the truthfulness of those details, to make these judgments? If so, how accurate are these judgments? These are the questions at the heart of our study.

How we make such judgments can be broadly described as * theory of mind*, or how we reason about the mental states of others based on their behavior (Premack & Woodruff, 1978). There is a wealth of research in this area, exploring issues from how we predict others’ intentions (e.g., Luchkina, Sommerville, & Sobel, 2018; Meltzoff , 1995), to how we understand false beliefs (e.g., Baron-Cohen, Leslie, & Frith, 1985; Gopnik & Astington, 1988; Wimmer & Perner, 1983) and the challenges of developing theory of mind in artificial intelligence (Aru, Labash, Corcoll, & Vicente, 2023). However, most of this research concerns how we reason about goal-directed behavior, not the extent of crystallized knowledge or the influence of environmental factors (Gweon, 2021). Further, scarce work in this area explores how people make judgments about others’ domain knowledge based on language alone. Although some developmental studies have explored this topic (e.g., Harris, Koenig, Corriveau, & Jaswal, 2018; Landrum & Mills, 2015; Lutz & Keil, 2002), there are relatively few incorporating adults.

Previous research has investigated the types of linguistic cues that distinguish experts from novices in knowledge communication. For example, experts have been shown to use more proper names (Isaacs & Clark, 1987), provide more suggestions and advanced language when giving advice (Levari, Gilbert, & Wilson, 2022; Hinds, Patterson, & Pfeffer, 2001; Reyt, Wiesenfeld, & Trope, 2016) and use more relational terms and a higher word-per-sentence ratio (Kim, Bae, Nho, & Lee, 2011). Moreover, people appear to use relatively simple linguistic cues when judging others. People can accurately distinguish the advice of medical doctors from those of lay people based on the length of words, with longer words used to infer expertise (Toma & D’Angelo, 2015). When taking advice from others, perceived usefulness is related to the amount of advice and the level of abstraction (Reyt et al., 2016; Levari et al., 2022) even if some of those linguistic cues are not diagnostic of the impact of advice on performance (Hinds et al., 2001).

In this research, we investigate people’s inferences about the knowledgeability of other people. In contrast to previous studies that involve tasks that require people to explain how to perform a task, such as playing a game, solving computer programming problems, or building electronic circuits (Levari et al., 2022; Hinds et al., 2001; Reyt et al., 2016), we use an image description task that requires people to produce explicit knowledge about referents in the image. We will investigate two types of cues to predict how people perceive the knowledgeability on the basis of these descriptions.

First, we investigate to what degree shallow cues related to *specificity* of language can be used to assess their domain knowledge. By the specificity of language, we refer to linguistic cues that point to knowledge of specific details that are not based on generic descriptions of the image. One type of specificity cue is the use of proper names (Isaacs & Clark, 1987). When people give proper names (for example, “Stephen King”, “Orlando Bloom”, “Philadelphia Warriors” in Fig. 1), it suggests that they know the names of the referents in the images. However, when describing images, people also elaborate on referents through statements that do not include proper names. For example, in Fig. 1, stating that Legolas the Elf is a “member of the fellowship of the ring”, or that Stephen King “generally writes horror books” suggests additional knowledge about the referents, but not in the form of proper names. Therefore, in this paper, we will go beyond the use of proper names and analyze the effect that any type of specific information has on perceived knowledgeability.

Second, we investigate how information related to the *veracity* of statements affects the perception of knowledgeability. Unlike cues related to specificity, information about veracity goes beyond language and requires explicit knowledge. Therefore, a key question is whether it takes an expert to accurately gauge the expertise of others. As domain experts can better discriminate between true and false statements in their domain, do they use the veracity in other people’s descriptions to assess knowledge? In contrast, do people who lack domain knowledge simply use the degree of specificity in other people’s language regardless of whether the statements that other people make are true or false? For example, the specific statement that the person depicted in Fig. 1b is Stephen King is true, but the statement that Stephen King wrote Jurassic Park is false. Similarly, the specific statement that the person depicted in Fig. 1b is Woody Allen is false (it is Martin Scorsese). Do these additional false details in these descriptions add to the perceived knowledgeability when the evaluator is less knowledgeable?

## Experiments 1 and 2

We explore these questions in two experiments. In Experiment 1, participants complete a series of general knowledge questions that span several categories, some of which they identify as being proficient, and others are randomly assigned. We refer to their score within each of these categories as their domain “knowledgeability”. They also provide written descriptions detailing the contents of various images representing these categories (Fig. 1 shows examples of such descriptions). In Experiment 2, a new set of participants (henceforth, “evaluators”) predict the relative knowledgeability of Experiment 1’s participants (henceforth, “informants”). We did this by having evaluators make pairwise comparisons between two informants’ image descriptions, selecting which informant they believe to be more knowledgeable. We varied the number of image descriptions displayed, assessing whether more information is helpful in estimating knowledgeability. Furthermore, we had the evaluators complete the same general knowledge task from Experiment 1 to assess whether their own knowledge predicted their ability to select the most knowledgeable informants. Critically, we investigated how evaluators at different levels of domain knowledgeability are influenced by the veracity of written descriptions when judging informants’ knowledge. We tested whether more knowledgeable evaluators discount informants who produce more false statements in their written descriptions.

Lastly, we tested whether the veracity of written descriptions influences evaluators’ decisions when making pairwise comparisons regarding informants’ knowledge. While some work has examined how variables such as time pressure affect our ability to judge the veracity of information (Sultan et al., 2022), it is unclear how the veracity of statements themselves affects the perceived credibility of their source.

## Methods

### Participants

A total of 100 informants were recruited for Experiment 1 and 160 evaluators were recruited for Experiment 2, all through Amazon Mechanical Turk (MTurk). To be eligible for our study, participants were required to have Master’s level status on MTurk, be United States residents, and be at least 18 years of age. The sample sizes were chosen to achieve a minimum number of ten participants in each of the general knowledge categories (see the Online Supplement for details).

### Materials

Nine hundred multiple-choice questions were adopted from a large corpus of trivia questions provided by a trivia question publisher (*The Question Co.*). These questions span 30 categories (e.g., Video Games, Biology, World Cup). Each question has four alternative fixed-choice responses. A total of 360 images were compiled for image description and informant discrimination trials. There are 12 images for each of the above-mentioned categories. Figure 1 demonstrates some of these images and participant descriptions.

### Procedure

Informants were presented the list of categories and instructed to select two in which they felt the most knowledgeable. Two additional categories were then randomly assigned. This was done to increase individual differences in knowledgeability scores. Throughout Experiment 1, informants completed two different tasks: describing images and answering general knowledge questions.

For image description trials, six images were simultaneously presented on-screen with open response fields below each one. Informants were instructed to provide multiple-word descriptions for each of the images, revealing any specific knowledge they had about the content depicted therein. If they lacked specific knowledge, they were asked to describe the image in general terms. Informants were explicitly discouraged from using external sources to look up answers (e.g., Wikipedia). If they deviated from the page (e.g., by opening another tab), they received a warning. After three warnings, they were removed from the study. There were eight such trials of this, two for each category. In total, each informant provided 48 image descriptions.

For the general knowledge question portion of our study, informants completed two blocks of 15 questions per category. They were given 20 s to complete each question. A countdown timer on the upper-right corner of the screen indicated this, turning red when 5 s remained. If no response was selected at this time, the choice buttons were locked and the informant was forced to proceed to the next question. No feedback was provided. At the end of each block, informants were asked to estimate how many of these 15 questions they answered correctly. In total, each informant completed 120 multiple-choice questions.

In sum, for each category, each informant produced 12 image descriptions, 30 multiple-choice answers, and two performance estimations.

Half of Experiment 2’s procedure followed that of Experiment 1, with evaluators selecting and being assigned categories to complete multiple-choice questions form. The key difference is in the image description portion of the experiment. In Experiment 1, informants provided descriptions of the images. In Experiment 2, evaluators assessed informants’ knowledge based on their image descriptions from Experiment 1.

Evaluators were presented with images and corresponding descriptions provided by pairs of informants. For a particular pair of informants, evaluators predicted which of the two informants was likely to perform better on the multiple-choice portion of the category depicted. The pairs of informants were randomly selected from Experiment 1 with the constraint that the informants had different accuracy scores on the multiple-choice questions of the category. There were eight prediction trials per category, with each trial involving a different pair of informants. The informants were labeled with distinct letter pairs (A-B, C-D, etc.). The number of images and corresponding descriptions presented varied (1, 3, 6, or 12) across trials. Overall, each evaluator answered 32 prediction trials.

For the general-knowledge questions in Experiments 1 and 2, participants provided a probability confidence rating for each response (25% (“Guessing”), 40%, 55%, 70%, 85%, 100% (“Absolutely Certain”)). For the evaluator discrimination task in Experiment 2, participants provided ordinal confidence ratings (“Very Confident,” “Somewhat Confident,” and “Guessing”) in the discrimination task. These confidence ratings were collected for the purpose of future studies on metacognition that relate confidence to accuracy but are not analyzed in the current study.

### Scoring of specificity of statements in image descriptions

The set of 4812 image descriptions was scored for specificity and veracity by a group of 21 raters. The full set of descriptions was first split into three smaller sets, with each set being scored by seven different raters. The raters were naive to the experiment’s methods and research questions. First, the raters were asked to extract specific statements from each image description (the exact instructions can be found in the data repository for this paper). These are statements that provide specific information about the referent in the image that is not based on generic descriptions of the content depicted or information already stated in the prompt or image itself. For example, the image description “Stephen King, the writer of Jurassic Park” by person C in Fig. 1b has two specific statements: “Stephen King” and “writer of Jurassic Park”. On the other hand, the image description “Orlando Bloom - long hair” by person F only has a single specific statement: “Orlando Bloom”. The additional statement that this person has “long hair” does not provide specific information that goes beyond what is visible in the image.

For each description, we counted the total number of specific statements for each rater. Table 1, row “Any”, shows the inter-rater reliability for each of three groups of raters as assessed by intraclass correlation coefficient (ICC). Rater agreement varies from moderate (e.g., 0.68) to good (0.82). For the purpose of data analysis, we use the mode of the number of specific statements across the raters. Therefore, for each image description, the degree of specificity is assessed by a single count.

The Online Supplement shows results for additional measures for specificity based on natural language processing techniques, including proper names and concreteness (Yeomans, 2021).

### Scoring of veracity of statements in image descriptions

The same set of raters also scored the veracity of image descriptions. For each specific statement that a rater annotated, the rater assessed whether the statement was true or false. A statement was scored as *true* when it accurately represented the content of the image. A statement was scored as *false* when it inaccurately represented the content of the image. For example, the statement “Stephen King” by Persons C and D in Fig. 1b correctly identifies the author pictured and is scored as a true statement. Conversely, the statement “wrote Jurassic Park” by Person C is scored as false: Jurassic Park was written by Michael Crichton and not Stephen King. To help facilitate the research to conduct the rating process, raters had access to the images as well as links to Wikipedia articles that contained information about the specific images being described.

For each image description and rater, we assessed veracity by counting the number of true and false specific statements. Table 1 shows the inter-rater reliability for these counts for each of three groups of raters as assessed by intraclass correlation coefficient (ICC). The rater agreement varies from moderate (e.g., 0.59) to good (e.g., .85) with a higher agreement on the counts of the number of true statements than the number of false statements. For our analyses, we summarize the results by the mode across the seven raters. Therefore, for each description, we have a single count of the number of true statements and a single count of the number of false statements.

**Table 1:** Inter-rater reliability, assessed by intraclass correlation coefficient (ICC) across three groups of raters

Statement type	Group 1	Group 2	Group 3
True	0.85	0.74	0.82
False	0.61	0.59	0.71
Any	0.82	0.68	0.79

The ICCs are provided for the raters’ counts of the number of true statements, the number of false statements, as well as the total number of specific statements

### Data analysis

For all analyses, we utilize Bayes factors (*BF*s) to determine the extent to which the observed data adjust our belief in the alternative and null hypotheses. Values of 3 < *BF* < 10 and *BF* > 10 indicate moderate and strong evidence against the null hypothesis, respectively. Similarly, values of 1/10 < *BF* < 1/3 and *BF* < 1/10 indicate moderate and strong evidence in favor of the null hypothesis, respectively (Jeffreys , 1961; Rouder, Speckman, Sun, Morey, & Iverson, 2009; Rouder, Morey, Speckman, & Province, 2012). In order to improve readability, *BF*s larger than 100 are reported as *BF* > 100. In addition to Bayes factors, we also report the 95% credible interval (CI) using Bayesian estimation methods. Although it might be tempting to use the CI to test hypotheses (e.g., rejecting the null hypothesis if the CI does not include the null value), in accordance with recent recommendations (van den Bergh, Haaf, Ly, Rouder, & Wagenmakers, 2021; Wagenmakers, Lee, Rouder, & Morey, 2020) we use a more conservative approach, where the CI becomes relevant only after the *BF* shows evidence for the alternative hypothesis.

For the Bayesian Pearson correlation and *t* tests, we computed the *BF* with the JASP software package (JASP Team, 2022) using the default priors that came with the software. For the logistic regression model, we applied Bayesian inference using a Markov chain Monte Carlo approach based on slice sampling. We ran the slice sampler with eight chains with a burn-in of 1000 iterations and took 100 samples from each chain after each 20th iteration. Convergence of the sampler was tested using standard methods. The *BFs* for the logistic regression model were performed using the Savage–Dickey method (Wagenmakers, Lodewyckx, Kuriyal, & Grasman, 2010). For all Bayesian analyses, we also performed *BF* robustness checks.

## Results

### Individual differences in knowledge

**Fig. 2:**
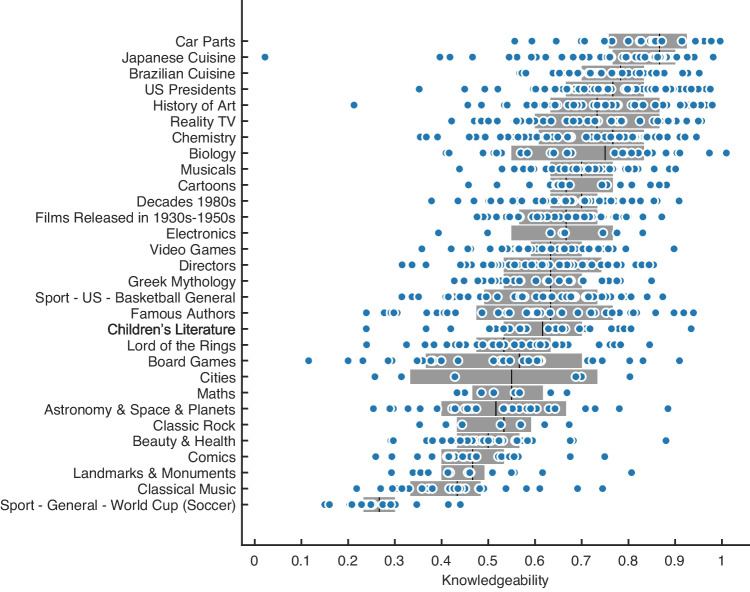
Individual participant accuracy across knowledge assessment categories. *Gray bars* show the 25–75% quartiles. Results are combined across participants in Experiments 1 and 2

Informants in Experiment 1 and evaluators in Experiment 2 showed substantial individual differences in knowledgeability within each category (Fig. 2). The mean accuracy difference between the worst and best participants in each category was .51 (average IQR is 0.19). In addition, participants were more accurate for categories that they self-selected as being knowledgeable in than for categories that were randomly assigned (*M* = .730 vs. *M* = .573, *BF* > 100).

### Knowledgeable informants produce more specific statements and fewer false statements

**Fig. 3:**
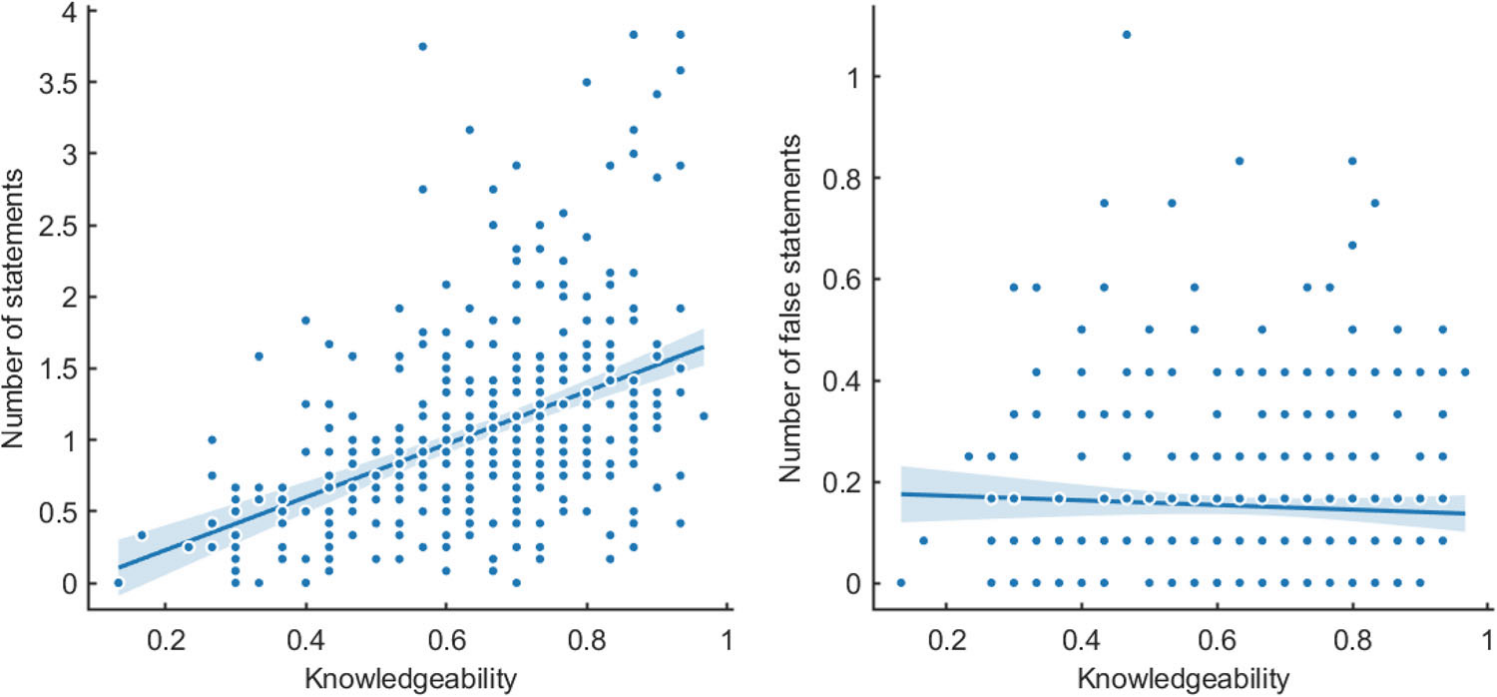
Number of statements (true or false) and number of false statements in the informant descriptions as a function of informant knowledgeability. *Lines* show linear regressions with 95% confidence intervals

Informants used a median of seven words (IQR = 4–12) in their descriptions. There was no evidence that more knowledgeable informants used more words overall (Pearson correlation *r* = 0.103, *BF* = .525, CI = [0.005,.199]) but informants used more words for the image descriptions of the categories they selected as their expertise relative to descriptions from categories that were randomly assigned to them (*M* = 9.9 vs. *M* = 8.8, *BF* = 11.8, CI = [8.5–11.3] vs. CI = [7.6–10]). Knowledgeable informants produced more specific statements (*r* = 0.453, *BF*> 100, CI = [0.370,.526]), as shown in Fig. 3, left panel. For example, informants who scored above 90% accuracy produced more than three times the number of specific statements as informants below 50% accuracy (*M* = 1.68 vs. *M* = .55). Overall, the results showed that knowledgeable informants did not necessarily produce longer descriptions but did use more specific language.

In terms of veracity, the likelihood that any specific informant statement within a description was false was low (*p* = .14) and therefore, the majority of informant statements within a description were true. Figure 3, right panel, reveals no evidence that knowledgeable informants produce fewer false statements overall (*r* = -.044, *BF* = .092, CI = [-0.141,0.054]). However, this analysis obscures differences across categories as well as images. Note that in Experiment 2, evaluators are comparing informant descriptions from the same image (which also belong to the same category). To investigate how informative the number of specific statements as well as the number of false statements are in discriminating between informants’ knowledgeability, we apply a logistic regression model to all pairwise informant comparisons in Experiment 2. The model includes as factors the difference in the total number of specific statements from informants A and B (regardless of whether they are true or not), as well as the difference in the total number of false statements from A and B. These totals are calculated across all the image descriptions provided by informants A and B on a particular trial. This leads to the regression model:

1 $$\begin{aligned}&p(A \, \text {is more knowledgeable than} \, B ) \nonumber \\&= f\left( w_0 + w_1 ( n_A - n_B ) + w_2 ( m_A - m_B ) \right) . \end{aligned}$$

where *f* is the logistic function, $n_A - n_B$ is the difference in the total number of specific statements made by A and B, and $m_A - m_B$ is the difference in the number of false statements.

**Fig. 4:**
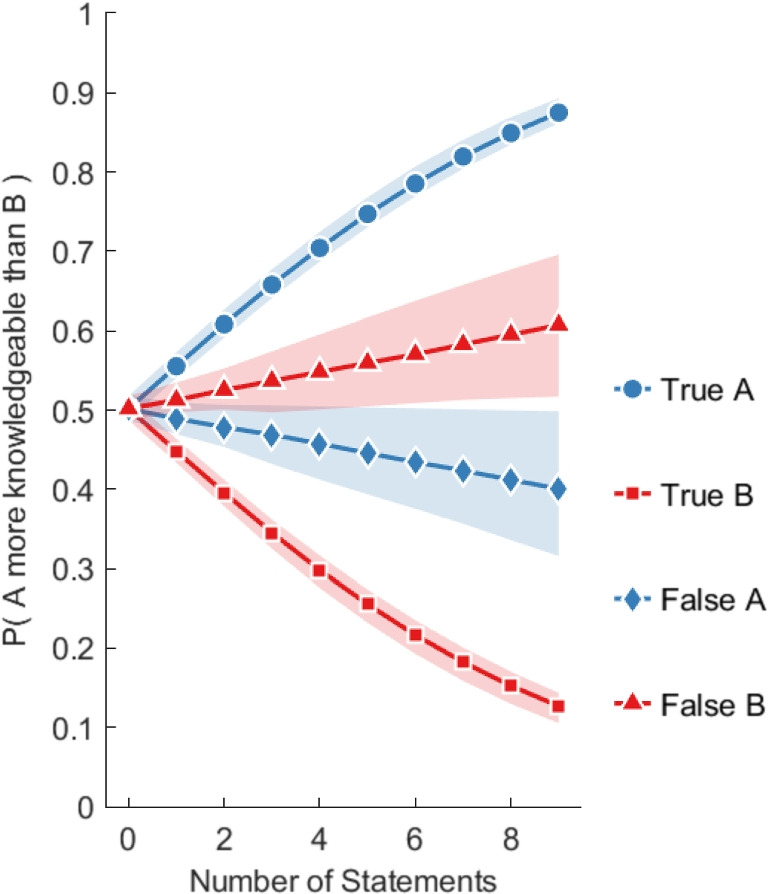
The estimated likelihood that informant A is more knowledgeable than B as a function of the estimated independent effects of the number of true and false statements by A and B. *Colored areas* represent 95% confidence intervals. Estimates were derived from the model in Eq. 1

We applied Bayesian inference to estimate model parameters and also performed a Bayesian model comparison with simpler models that excluded each individual term. The model comparison results show that the model that includes each of the individual factors ($w_1$ and $w_2$) is significantly better at describing the data than a model that removes one of those factors (*BF* > 100). The posterior mean of $w_1$ was positive (*M* = .22, CI = [.20,.24]), while the posterior mean of $w_2$ was negative (*M* = -.26, CI = [-0.30,-0.22]). To facilitate the interpretation of the estimated model, Fig. 4 visualizes how the number of statements that are true and false from A and B independently affect the likelihood that A is more knowledgeable than B. The results show that increasing the number of true statements by A, and decreasing the number of false statements by B makes it more likely that A is more knowledgeable. However, the estimated effect of the number of false statements is smaller in comparison to the number of true statements. Overall, the modeling results show that the number of specific statements by informants provides a useful indication of their knowledge while the number of false statements provided a more subtle cue for knowledge.

### Factors influencing evaluator accuracy

When presented with image descriptions from pairs of informants, evaluators were generally above chance in determining the most knowledgeable informant. With a single image description, evaluator accuracy was 65%. To put this level of performance into context, it should be noted that the ground truth is based on the score from a limited set of 30 multiple-choice questions. To get an estimate of the upper limit in performance we can expect in this discrimination task, we used a split-half reliability procedure tailored to the discrimination task. In this procedure, we estimated the accuracy that could be obtained if evaluators had knowledge of the number of items answered correctly by each pair of informants on a randomly chosen half of the questions, and then used this information to predict which informant would score higher on the remaining (unseen) half of the questions. The probability that this strategy leads to the correct prediction is 79%. Therefore, even with direct information about performance on half of the general knowledge questions, predictions are at most 79% accurate and this level of accuracy can serve as a useful performance ceiling to interpret the actual observed evaluation accuracy.

**Fig. 5:**
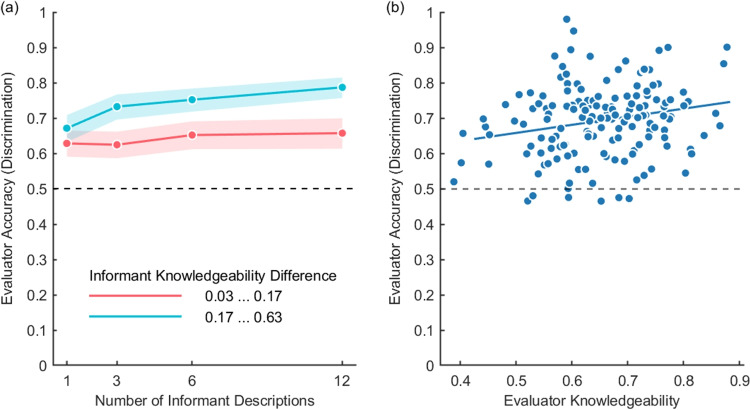
Evaluator accuracy in the discrimination task. **a** Mean evaluator accuracy for different number of informant descriptions and differences in informants’ knowledgeability, below (*red*) and above (*blue*) the median of differences. **b** Individual evaluator accuracy as a function of their knowledgeability. *Dashed lines* represent chance performance. *Colored areas* represent the 95% confidence interval

Evaluator accuracy increased as more descriptions from the same informants were presented to evaluators (Fig. 5a). With a single informant description, evaluator accuracy was 65%. Performance increased to 74% with 12 descriptions (*BF* > 100, paired-samples *t* test). Additionally, evaluators were more accurate when they had to discriminate between informants with larger differences in their knowledgeability (Fig. 5b). There is moderate evidence that more knowledgeable evaluators performed better in the discrimination task (Pearson *r* = .226, *BF* = 5.9, CI = [.073,.365]), but as illustrated in Fig. 5b, there are substantial individual performance differences across the two tasks.

### More knowledgeable evaluators make differential use of false statements relative to true statements

The results so far have established that more knowledgeable informants use more specific statements that are true and fewer specific statements that are false. The key question now is to assess to what extent evaluators are sensitive to this information and whether more knowledgeable evaluators make differential use of the available cues. In particular, we want to assess whether more knowledgeable evaluators are more likely to discount informants who express more false statements. To investigate these questions, we estimate a variant of the regression model in Eq. 1 but where the goal is to predict the choices that evaluators make:

2 $$\begin{aligned} p(\text {Choose}\,A ) = f\left( w_0 + w_1 ( n_A - n_B ) + w_2 \theta ( m_A - m_B ) \right) \end{aligned}$$

The model includes as factors the difference in the total number of specific statements from A and B (regardless of whether they are true or not), as well as the difference in the total number of false statements from A and B, as well as an interaction effect with the knowledgeability of the evaluator (denoted by $\theta$) to test if more knowledgeable evaluators are more sensitive to false statements.

**Fig. 6:**
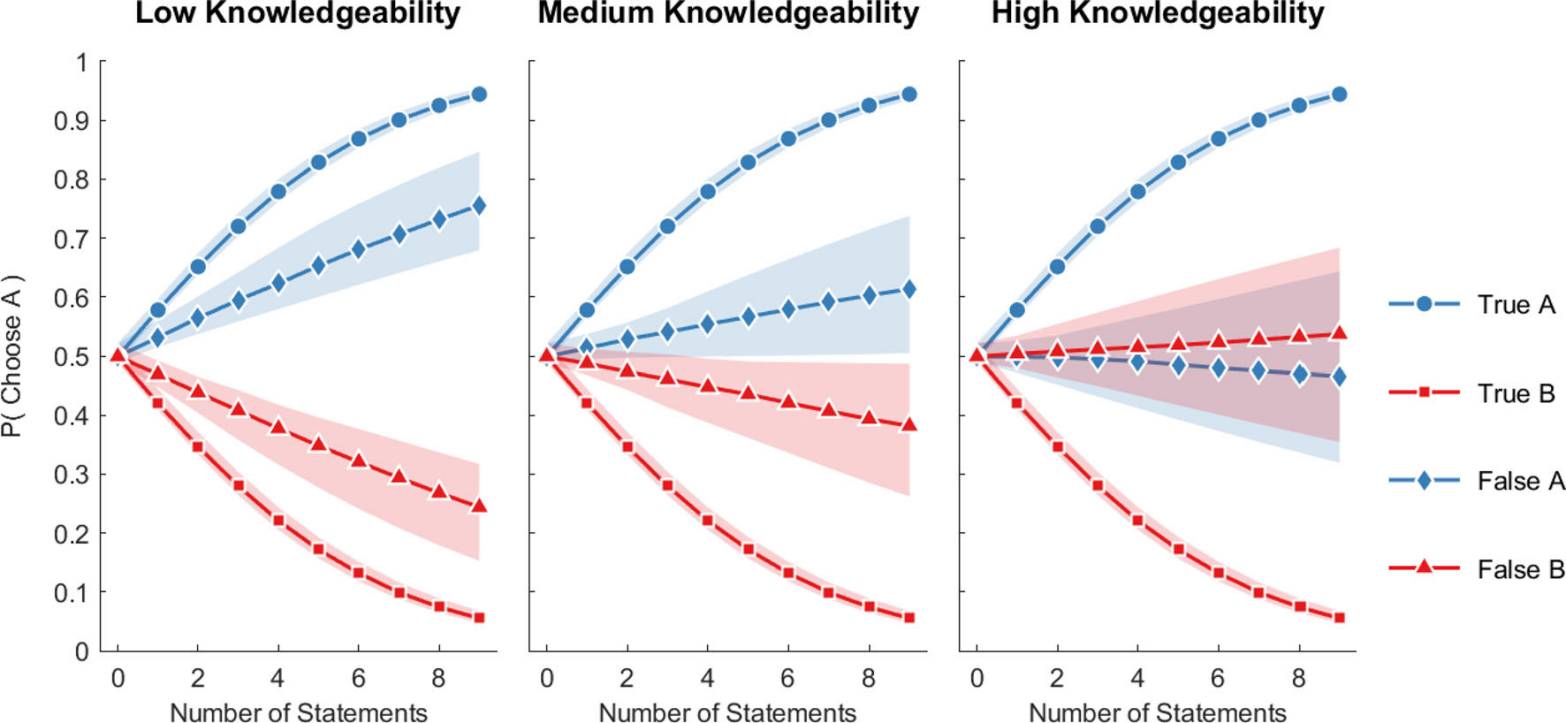
The estimated probability that an evaluator will choose informant A as the more knowledgeable person as a function of the estimated independent effects of the number of true and false statements from A and B. Results are separated by the knowledgeability of the evaluator at three values of $\theta$ (Low = 0.5, Medium = 0.75, and High = 0.9 accuracy). *Colored areas* represent 95% credible intervals. The estimates were derived from the logistic regression model in Eq. 2

We applied Bayesian inference to estimate model parameters and also performed a Bayesian model comparison with simpler models that excluded each individual term. The results of the model comparison show that there is evidence for the first factor involving $w_1$, as well as the interaction factor involving $w_2$, $(BF > 100)$. Therefore, the model that includes those factors is significantly better at describing the data than a model that has one of those factors removed. The posterior mean of $w_1$ was positive (*M* = .32, CI = [.29,.34]), while the posterior mean of $w_2$ was negative (*M* = -.36, CI = [-0.43,-0.29]). Therefore, if out of two informants A and B, A produces a larger number of specific statements (regardless of whether they are true or false), it becomes more likely that evaluators choose A as the most knowledgeable. Furthermore, if informant A produces more false statements, it becomes less likely that evaluators will pick informant A, but this tendency depends on the knowledgeability of the evaluator.

To facilitate the interpretation of the estimated model and especially the interaction between knowledgeability and the number of false statements, Figure 6 shows the model predictions when varying the number of true and false statements while keeping all other factors constant. The model predictions are separated by low, medium and high knowledge assessors. These results show that evaluators of *all* levels of knowledgeability tend to select informants who produce a higher number of true statements. The less knowledgeable evaluators tend to treat false statements more similarly to true statements – that is, increasing the number of false statements for informant A makes these evaluators *more* likely to choose informant A. However, for more knowledgeable evaluators, this effect is reversed, though not completely. Overall, these results demonstrate that evaluators are sensitive to the number of specific statements, but only the more knowledgeable evaluators tend to discount the effect of false statements.

The Online Supplement shows a different visualization of model predictions and empirical results. In addition, the Online Supplement shows results from an expanded regression model that adds additional factors, including the number of words, the number of proper nouns, and a measure of concreteness (Yeomans, 2021). The results of the expanded model are consistent with those of the model presented here.

## Discussion

Across two experiments, we addressed the fundamental question of whether a few words may provide accurate estimates of domain knowledge in others. We showed that the most knowledgeable informants produce the most specific statements, with those who perform above 90% accuracy in the general knowledge question task producing more than three times as many specific statements as those who perform below 50% accuracy. Additionally, evaluators at all knowledge levels select informants who produce more specific information. These two results are consistent with previous studies showing that experts provide more specific information in terms of suggestions (Levari et al., 2022) and proper names (Isaacs & Clark, 1987) and the amount of specific information affects the perceived usefulness of advice (Levari et al., 2022).

A key new result is that the veracity of statements only affects the most knowledgeable evaluators. Presumably, the more knowledgeable evaluators are able to differentiate true and false statements and downward adjust the perceived informant knowledgeability after encountering a particular detail that is false. Less knowledgeable evaluators are less likely to have the required domain knowledge to differentiate true statements from false ones, and are more readily swayed by specific information, regardless of its ground truth. Overall, our results show that relatively low-level linguistic cues are used to evaluate other people’s knowledge when the knowledge is outside the evaluator’s domain of expertise. Only when the evaluator is knowledgeable in a domain can they go beyond these simple strategies to also consider the veracity of the information provided separate from the amount of information.

One limitation of our study is that the base rate of false statements was quite low, which restricted our ability to assess the effectiveness of any “fact checking” strategies used by evaluators. Future work should investigate whether our findings persist when the presence of false statements matches − or *exceeds* − their true counterparts. This may be done through experimental manipulation or naturalistic means. This approach should further reveal whether the specificity of statements is a useful heuristic in determining others’ knowledge.

A natural extension of this work is to examine how domain experts and novices treat misinformation differently. This is an obvious and growing concern not only on social media platforms, such as Twitter (e.g., Kouzy et al. , 2020), but also in textual information provided by generative AI platforms. For example, responses from recently developed generative search engines often appear fluent and informative, but on further investigation are frequently found to contain unsupported statements and inaccurate citations (Liu, Zhang, & Liang, 2023). Heuristics that rely on simple linguistic features, such as fluency and the amount of detail provided, are likely to provide misleading cues about the actual usefulness of the information. As our results suggest that individual differences in evaluator knowledge lead to differential use of true and false statements, it is worth exploring how these individual differences contribute to the perceived usefulness of information in online environments. Another extension is to study effortful deception, such as having high-knowledge informants construct false statements and examining how effective others are at fact-checking them across the knowledge spectrum. Such approaches can help us better understand how false statements are evaluated and are a potentially crucial step in combating misinformation.

### Supplementary Information

Below is the link to the electronic supplementary material.

Supplementary file 1 (pdf 129 KB)

## Data Availability

All data and stimuli from this study are accessible on the Open Science Framework: https://osf.io/4q8ph/?view_only=20cd8ae26b2d44b18d340f7d7f15bf99. None of the experiments were preregistered.
