# Supplementary material for: How experts and novices judge other people’s knowledgeability from language use
Source: Psychon Bull Rev. 2024 Jan 4;31(4):1627–37. doi: 10.3758/s13423-023-02433-9 (PMC11358192; doi:10.3758/s13423-023-02433-9)

Supplement to: How Experts and Novices Judge Other People's Knowledgeability from Language Use

Sample size considerations

The primary rationale for the sample sizes of Experiments 1 and 2 was to ensure that a majority of the 30 categories of general knowledge would be available for data analysis. For Experiment 1, the goal was to obtain data from at least 10 participants in a majority of the 30 categories. Using a Monte Carlo analysis, assuming that the four categories (two by choice, two randomly assigned) have equal probability of being chosen, we established that with a sample size of 100 participants there is a 95% probability that at least 24 of the 30 categories would meet the criterion number of participants (with 80 participants, this drops to 17 out of 30 categories). For Experiment 2, we also aimed to have at least 10 participants in a majority of categories, but faced the additional constraint that these had to be the same categories as in Experiment 1. Through a Monte Carlo analysis, assuming the same sampling process as for Experiment 1, we established that with a sample size of 160 participants in Experiment 2, there is a 95% probability of obtaining at least 24 of 30 categories with at least 10 participants in *both* Experiment 1 and 2 (for comparison, with 100 participants in Experiment 2, this drops to 20).

Results with other measures of specificity

The main article focuses on language specificity as assessed by the number of specific statements in an image description. This measure requires a procedure in which raters manually annotate each specific statement from a description. Here, we consider the use of natural language processing (NLP) techniques that can automatically extract useful statistics from descriptions. Specifically, we focus on the use of proper nouns that have previously been associated with expert knowledge (Isaacs and Clark, 1987). In addition, we also investigate concreteness, a word-level NLP measure that has been used before to analyze people's advice giving and planning processes (Yeomans, 2021). To assess concreteness, we used the *doc2concrete* software package with default settings.

Table 1 shows the correlations between these NLP measures, the number of specific statements, and the number of words. Not surprisingly, the number of proper nouns correlates with the number of specific statements, as specific statements include those mentioning proper names. However, note that the count of specific statements included not only proper names, but also any other statements that referenced detailed information not in the form of names of people, places, or things. Therefore, the number of specific statements is a larger set of statements than the set of statements involving proper names. The number of specific statements also correlates with the number of words, although the correlation is weak.

The concreteness measure does not correlate well with the other measures, suggesting that this measure picks up aspects of language use that are different from providing details about referents in the images.

Table 1: Correlation between four linguistic measures related to specificity

	#Specific statements	#Proper Nouns	Concreteness	#Words
#Specific statements	+1.00	+0.62	-0.24	0.37
#Proper Nouns	+0.62	+1.00	-0.32	0.30
Concreteness	-0.24	-0.32	+1.00	-0.00
#Words	+0.37	+0.30	-0.00	+1.00

It should also be noted that the *doc2concrete* function that implements the concreteness measure is not designed to handle proper names that frequently appear in our specific statements. For example, in our testing of this measure, we found that adding more proper names to a description *lowers* the concreteness value while replacing proper names by generic descriptions *increases* the concreteness value, effects that are opposite to our specificity measure.

An expanded regression model to explain the relative knowledge of the informant

The main paper describes a regression model in which the goal is to predict which of two informants is more knowledgeable. Here we expand the model to also include the number of words, the number of proper nouns, and concreteness as additional factors:

$$p(\text{A is more knowledgeable than B }) = f(w_0 + w_1(n_A - n_B) + w_2(m_A - m_B) + w_3(d_A - d_B) + w_4(e_A - e_B) + w_5(f_A - f_B)) \tag{1}$$

In this equation, f is the logistic function, $n_A - n_B$ is the differential in the number of specific statements made by A and B, $m_A - m_B$ is the differential in the number of false statements, $d_A - d_B$ is the differential in the number of words (expressed in logarithmic units), $e_A - e_B$ is the differential in the number of proper nouns, and $f_A - f_B$ is the differential in concreteness.

Bayesian model comparisons show that there is support for including each factor in the model ($BF > 100$) except for w_3 associated with word count (BF= 0.04). The posterior mean of w_1 is positive (M = 0.14, CI = [0.12,0.17]), while the posterior mean of w_2 is negative (M = -0.27, CI = [-0.32,-0.23]). These results are similar to the results described in the main paper, where only these factors were included in the model, and show that a more knowledgeable informant produces more specific statements overall and fewer false statements. Furthermore, these results show that the additional NLP features do not change the interpretation of the model.

For the additional factors that we added that have significant support, the posterior mean of w_4 is positive (M = 0.07, CI = [0.05,0.08]) and the posterior mean of w_5 is negative (M = -0.35, CI = [-0.43,-0.27]). Therefore, out of a pair of two informants, the more knowledgeable informant tends to use more proper nouns and less concrete language as assessed by the concreteness NLP measure while the number of words used is not predictive. Note that the direction of the concreteness effect should be treated with caution as the concreteness measure is not designed to handle proper names (see section "Results with other measures of specificity").

To compare the effects of the different measures for specificity, we looked at the absolute value of weights when the regression model is estimated with standardized inputs (mean centered and unit standard deviation). The effect of the number of specific statements is greatest, followed by proper nouns with concreteness having the smallest effect ($|w_1|$=0.93, $|w_4|$=0.64, and $|w_5|$=0.27, respectively).

An expanded regression model to explain the choices of the evaluators

The main paper describes a regression model in which the goal is to predict the choices the evaluators make. Here we expand the model to also include the number of words, the number of proper nouns, and concreteness as additional factors:

$$p(\text{Choose } A) = f(w_0 + w_1(n_A - n_B) + w_2\theta(m_A - m_B) + w_3(d_A - d_B) + w_4(e_A - e_B) + w_5(f_A - f_B)) \tag{2}$$

The same factors are used as in Eq. 1 but with an interaction effect with the knowledgeability of the evaluator θ. Bayesian model comparisons show that each factor is significant ($BF > 100$) except w_3 associated with the word count (BF=0.44). The posterior mean of w_1 is positive (M = 0.25, CI = [0.23,0.28]), while the posterior mean of w_2 is negative (M = -0.36, CI = [-0.42,-0.29]). These results closely mirror the results described in the main paper where only these factors were included in the model and show that the additional word measures do not change the interpretation of the model.

For the additional factors that we added that have significant support, the posterior mean of w_4 is positive (M = 0.05, CI = [0.04,0.06]) and the posterior mean of w_5 is negative (M = -0.32, CI = [-0.39,-0.23]). Therefore, if informant A uses more proper nouns in their descriptions and uses less concrete language

(as assessed by the concreteness measure), it becomes more likely that evaluators pick informant A. The number of words produced by an informant is not predictive of evaluators' choices. As mentioned before, the direction of the concreteness effect should be treated with caution, as the concreteness measure is not designed to handle proper names (see section "Results with other measures of specificity").

To compare the effects of the different measures for specificity, we looked at the absolute value of weights when the regression model is estimated with standardized factors (mean centered and unit standard deviation). The effect of the number of specific statements is greatest, followed by proper nouns with concreteness having the smallest effect ($|w_1|$=1.63, $|w_4|$=0.46, and $|w_5|$=0.24, respectively).

Overlaying empirical results for the choice model

We visualize the predictions of the model described in Eq. 2 of the main paper to allow for a comparison between the model predictions and the empirical data.

Figure 1 shows the observed probability (top row) and estimated probability (bottom row) that an evaluator will choose informant A as the more knowledgeable person as a function of the difference in the number of specific statements regardless of truth (left column) and incorrect statements (right column). The results are separated by the knowledgeability of the evaluator (Low: $\theta < 0.7$; High: $\theta > 0.7$). The estimates were derived from the logistic regression model in Eq. 2. The data show that evaluators are more likely to choose A over B as the most knowledgeable informant if informant A has more specific statements. Only highly knowledgeable evaluators use information about truthfulness in their choices and are *less* likely to choose informant A over B if informant A makes more false statements than B. The model captures this interaction between knowledgeability and the differential of the number of false statements. The model does not capture the interaction between knowledgeability and the differential of the total number of specific statements. This would require adding additional terms to the regression model.

References

Isaacs, E. A. and Clark, H. H. (1987). References in conversation between experts and novices. *Journal of Experimental Psychology: General*, 116(1):26.

Yeomans, M. (2021). A concrete example of construct construction in natural language. *Organizational Behavior and Human Decision Processes*, 162:81–94.

Figure 1: The observed and estimated probability that an evaluator will choose informant A as the more knowledgeable person. The model predictions are derived from fits of the regression model in Eq. 2

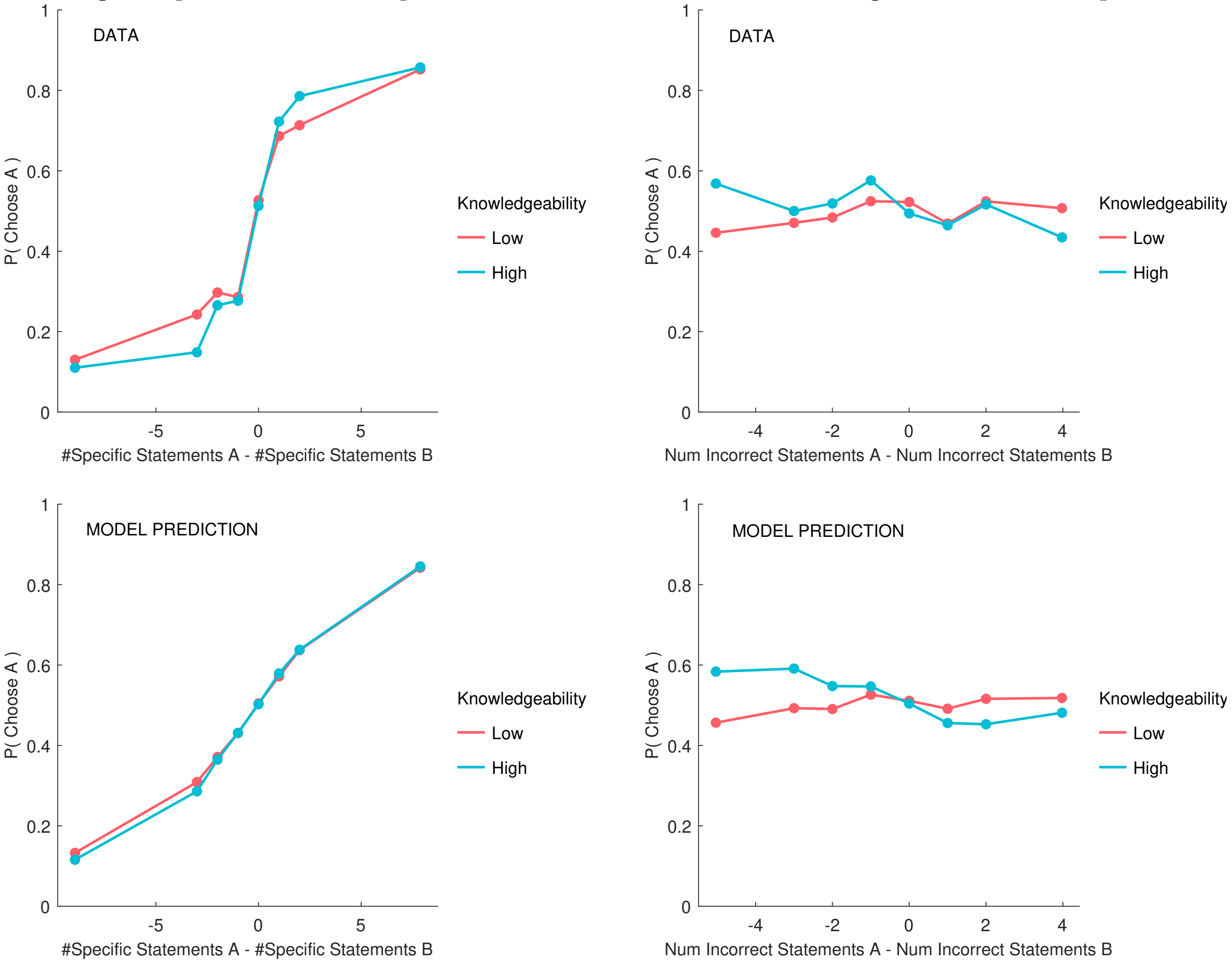

Supplement: Supplementary file 1 — (pdf 129 KB) [file 13423_2023_2433_MOESM1_ESM.pdf]
